# Primary Graft Function and 5 Year Insulin Independence After Pancreas and Islet Transplantation for Type 1 Diabetes: A Retrospective Parallel Cohort Study

**DOI:** 10.3389/ti.2023.11950

**Published:** 2023-12-28

**Authors:** Mikael Chetboun, Christophe Masset, Mehdi Maanaoui, Frédérique Defrance, Valéry Gmyr, Violeta Raverdy, Thomas Hubert, Caroline Bonner, Lisa Supiot, Clarisse Kerleau, Gilles Blancho, Julien Branchereau, Georges Karam, Ismaël Chelghaf, Aurélie Houzet, Magali Giral, Claire Garandeau, Jacques Dantal, Kristell Le Mapihan, Arnaud Jannin, Marc Hazzan, Robert Caiazzo, Julie Kerr-Conte, Marie-Christine Vantyghem, Diego Cantarovich, François Pattou

**Affiliations:** ^1^ Univ Lille, U1190 - EGID, Lille, France; ^2^ Inserm, U1190, Lille, France; ^3^ Institut Pasteur de Lille, Lille, France; ^4^ CHU Lille, Department of General, Endocrine and Metabolic Surgery, Lille, France; ^5^ Institut de Transplantation Urologie Néphrologie (ITUN), Service de Néphrologie et Immunologie clinique, CHU Nantes, Nantes, France; ^6^ Nantes Université, Inserm, UMR 1064, Center for Research in Transplantation and Translational Immunology, Nantes, France; ^7^ CHU Lille, Department of Nephrology, Lille, France; ^8^ CHU Lille, Department of Endocrinology, Diabetology and Metabolism, Lille, France

**Keywords:** insulin independence, prediction, islet transplant, pancreas allograft, primary graft function

## Abstract

In islet transplantation (ITx), primary graft function (PGF) or beta cell function measured early after last infusion is closely associated with long term clinical outcomes. We investigated the association between PGF and 5 year insulin independence rate in ITx and pancreas transplantation (PTx) recipients. This retrospective multicenter study included type 1 diabetes patients who underwent ITx in Lille and PTx in Nantes from 2000 to 2022. PGF was assessed using the validated Beta2-score and compared to normoglycemic control subjects. Subsequently, the 5 year insulin independence rates, as predicted by a validated PGF-based model, were compared to the actual rates observed in ITx and PTx patients. The study enrolled 39 ITx (23 ITA, 16 IAK), 209 PTx recipients (23 PTA, 14 PAK, 172 SPK), and 56 normoglycemic controls. Mean[SD] PGF was lower after ITx (ITA 22.3[5.2], IAK 24.8[6.4], than after PTx (PTA 38.9[15.3], PAK 36.8[9.0], SPK 38.7[10.5]), and lower than mean beta-cell function measured in normoglycemic control: 36.6[4.3]. The insulin independence rates observed at 5 years after PTA and PAK aligned with PGF predictions, and was higher after SPK. Our results indicate a similar relation between PGF and 5 year insulin independence in ITx and solitary PTx, shedding new light on long-term transplantation outcomes.

## Graphical Abstract

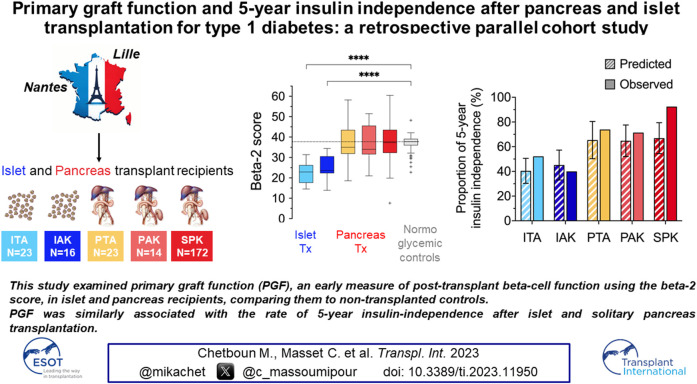

## Introduction

Type 1 diabetes is caused by the autoimmune destruction of pancreatic beta-cells, leading to a complete deficiency of insulin secretion [1]. While exogenous insulin therapy remains the standard treatment, allogeneic transplantation of either whole pancreas organs or isolated pancreatic islets have emerged as validated therapeutic approaches in patients with severe forms of type 1 diabetes (T1D). The choice between pancreas (PTx) or islet transplantation (ITx) depends on various factors, including recipient characteristics, risk of immunosuppressive regimen and associated comorbidities [2–6].

In PTx, the vascularized transplanted organ rapidly restores endogenous insulin production, resulting in a substantial improvement in glycemic control, sustained insulin independence over years and the potential for regression of diabetic degenerative complications, including nephropathy lesions [7]. In patients with end stage renal failure, simultaneous pancreas-kidney transplant (SPK) was also linked to enhanced patient survival [8]. On the other hand, the transplantation of a vascularized pancreas requires a major surgical procedure which carries specific risks, such as bleeding, infection, and vascular thrombosis. Stringent patient selection is therefore crucial to minimize risks and ensure successful outcomes [9–11].

ITx entails only a minimally invasive procedure consisting of the infusion of few milliliters of isolated pancreatic islets into the portal vein, typically using a radiological or mini-surgical approach [3, 12–14], resulting in limited risks.

Although partial islet graft function is sufficient to suppress severe hypoglycemia [15], multiple islet infusions are often required to achieve sustained insulin independence [16–18].

Overall, PTx results in better long-term metabolic results than ITx [19–22], with the best long term outcome being reported after SPK. The reasons underlying these discrepancies are not fully elucidated. Assessing and predicting long-term graft function is an important objective for optimizing patient outcomes.

In the field of ITx, long-term graft survival has been related to the early estimate of transplanted beta-cell function, also named primary graft function (PGF) [16, 23]. A recent global study analyzing 1210 islet recipients from the international Collaborative Islet Transplant Registry [17], confirmed this tight relation between primary graft function, estimated 1 month after last islet infusion with the Beta2-score, a validated index of beta-cell function [24], and the overall 5 year success of ITx. Importantly, this association was independent of graft characteristics such as the number of islet infusions, the total transplanted islet mass, and also of the immunosuppressive regimen. These findings designate primary graft function as a robust early endpoint, which can be used to predict long-term outcomes in ITx [17]. In contrast, the evaluation of primary graft function (within first weeks after surgery) and its relation with long-term success (i.e., insulin independence) has not been explored in PTx recipients.

The primary objective of the present study was therefore to analyze and compare the potential association of primary graft function estimated soon after transplantation, and the 5 year rate of insulin independence in patients receiving an ITx and for the first time in patients receiving PTx.

## Patients and Methods

### Study Design

This retrospective multicentre cohort study was designed to estimate primary graft function in patients who received beta-cell replacement with either PTx or ITx, and to analyze its relation with the rate of 5 year insulin independence. In addition, we also compared primary graft function in transplanted patients with beta-cell function estimated in non-transplanted normoglycemic individuals.

### Study Population

#### Pancreas and Islet Transplantation Recipients

We enrolled participants from two single-center cohorts of ITx and PTx in whom all variables required to calculate primary graft function were available within weeks after transplantation, and a follow-up of at least 5 years.

PTx was performed at Nantes University Hospital between 2000 and 2022. Recipients aged from 18 to 65 years old were included if they received pancreas transplantation alone (PTA), pancreas after kidney (PAK) or simultaneous pancreas and kidney transplantation (SPK), had a functional pancreas graft, and available variables to calculate the Beta-2 score (HbA1c mostly available after the third post-operative month), and a follow-up of at least 5 years. Procurement of pancreases, for both PTx and ITx, was obtained from ABO-compatible/MHC-unmatched brain-dead deceased donors with a negative T-cell cross-match. Whole organ pancreas was transplanted following procurement (i.e., less than 12 h) using a duodeno-enteric anastomosis, either with or without Roux-en-Y. Portal or systemic venous diversion was performed. Kidney transplantation was performed according to standard surgical procedure [25]. The induction immunosuppressive strategy consisted of a T-cell depleting agent (anti-thymocyte globulin for 5 days) and TNF-alpha inhibitor Etanercept (since 2017), tacrolimus and antitiproliferative agent mycophenolate mofetil or mycophenolic acid, all at standard and recommended doses. Steroids were administered for only 7–10 days.

ITx was performed at Lille University Hospital between 2003 and 2017, as previously described [14]. Briefly, recipients were patients with C-peptide negative type 1 diabetes, aged from 18 to 65 years old who received an islet transplantation alone (ITA) or after kidney transplantation (IAK) in the context of three prospective trials (ClinicalTrials.gov Identifier: NCT00446264/NCT01123187 [16] and NCT01148680 [26]) and a follow-up of at least 5 years. Islets were isolated within 12 h following pancreas procurement and cultured for up to 72 h prior to transplantation [27]. ITx consisted of two to three sequential intraportal islet infusions within 3 months, with the aim of reaching adequate metabolic control (i.e., HbA1c ≤ 6.5% without severe hypoglycemia) without exogenous insulin. No re-transplantation was performed during the follow-up even when the patient had lost his islet graft. Access to portal vein was obtained under general anesthesia by percutaneous transhepatic catheterization of a peripheral portal branch under ultrasound guidance or by a surgical mini-invasive laparotomy with vascular approach of a proximal mesenteric vein. Heparin (35 units/kg of recipient body weight) was added to the final human islet preparation, gently infused by gravity with portal pressure monitoring as previously described [16, 23]. Participants received Interleukin-2 receptor antagonist (Daclizumab^TM^) induction with sirolimus and tacrolimus maintenance (trials NCT00446264 and NCT01123187) [16]. Participants from trial NCT01148680 [26] received induction with TNF-alpha inhibitor (Etanercept^TM^), T-cell depleting agent (anti-thymocyte globulin) for first infusion or with Interleukin-2 receptor antagonist for second or third infusions followed by maintenance therapy using tacrolimus and antiproliferative agents (mycophenolate mofetil).

#### Controlled Non-transplanted Population

In addition, we also analyzed data from normoglycemic adult individuals enrolled in two prospective cohorts (OBEDIAB, ClinicalTrials.gov Identifier: NCT00688974; and ABOS, ClinicalTrials.gov Identifier: NCT01129297), at Lille University Hospital between 2004 and 2022 for surgery. Participants with a body mass index comprised between 18 and 40 kg/m2 and normal glucose control (fasting plasma glucose <5.6 mM/L, 2 h plasma glucose <7.8 mM:L, HbA1c<5.7%), in whom the four variables required to calculate the Beta-2 score were available at the baseline visit, were included in the present study.

### Data Collection

Recipient, donor and transplantation characteristics were collected in the ITx and PTx cohort prior to transplantation. Including recipient age, sex, body mass index (BMI), pre-transplant glycemic status, immunosuppressive regimens, graft characteristics. The total islet mass transplanted was expressed in islet-equivalent (i.e., one islet-equivalent corresponds to the tissue volume of one spherical islet with a diameter of 150 μm [28]). Allogeneic immunization prior to transplantation was evaluated by complement-dependent lymphocytotoxicity assay prior to 2007 and by the LABScreen Mixed Luminex flow bead assay (One Lambda^TM^) after 2007 and preformed donor-specific antibodies (DSA) were defined as positive if minimum mean fluorescent intensity (MFI) was equal to or greater than 500 in ITx and 1000 in PTx recipients.

Study protocols were approved by the Institutional Review Board and were previously published [16, 23, 25, 26, 29]. PTx data were extracted from the French Nantes DIVAT cohort approved by the French CNIL (n°914184). The quality of the DIVAT data bank is validated by an annual cross-center audit and has been reviewed by the appropriate ethics committee in accordance with the ethical standards laid down in the Declaration of Helsinki 2000 as well as the Declaration of Istanbul 2008. The database was locked on July 1, 2023. The implementation of the database refers to the standard operating procedures established in accordance with the European Data Protection Directive (95/46/EC) and, upon its entry into force, Regulation (EU) 2016/679, also referred as the General Data Protection Regulation (GDPR), with the French CNIL concerning the processing of personal data in clinical studies. Data were de-identified before analysis in order to respect confidentiality. A signed informed consent was obtained from all ITx, PTx and OBEDIAB/ABOS patients.

### Exposure of Interest

The study exposure of interest was primary graft function, an early estimation of the functional beta-cell mass after transplantation. In ITx, primary graft function was assessed as previously described, 1 month after the last islet infusion (2–6 months after first islet infusion) [16, 17]. In PTx, since HbA1c level was rarely measured before the end of the third month after surgery, primary graft function was assessed at this time period. In all cases, primary graft function was estimated with the Beta-2 score, a continuously validated variable (in which 0 represents no beta-cell function) calculated using a fasting blood sample based on values of fasting C-peptide (nmol/L), fasting blood glucose (mmol/L), HbA1c (%), and daily exogenous insulin needs per kg of body weight (IU/kg per day) [24]. In the OBEDIAB/ABOS cohort, beta-cell function was similarly estimated with the Beta-2 score using the fasting values of C-peptide, blood glucose, and HbA1c measured during a 75 g oral glucose tolerance test prior to surgery and allowed to classify the glucose tolerance disorder of each patient according to the criteria of the American Diabetes Association. In this population only normoglycemic controls were included in the present study.

### Outcome

The success of transplantation was defined as insulin independence, i.e., no exogenous insulin needs for a minimum of 14 consecutive days, assessed 5 years after transplantation.

### Statistical Analysis

Quantitative variables were expressed as means ± standard deviation in cases of normal distribution or medians (interquartile range, IQR) otherwise. Categorical variables were expressed as numbers (percentage). Normality of distributions was assessed using histograms and the Shapiro-Wilk test.

Pre-transplant recipient and transplantation characteristics were described for three different subgroups: ITA/IAK, PTA/PAK, and SPK. Beta-2 score and its determinants, fasting serum C-peptide, and HbA1c, were described for different subgroups in ITx, PTx recipients, and in OBEDIAB/ABOS individuals and continuous variables were compared using the One-way Welch ANOVA test. Note that only patients with a functional pancreatic graft at 3 months were analyzed in this study (per-protocol analysis, excluding patients with primary graft failure), whereas all islet-transplanted patients had a functioning graft at 1 month and were included in the analysis (intention-to-treat analysis).

For each subgroup of recipients (ITA, IAK, PTA, PAK, and SPK), we calculated the mean observed 5-year rate of insulin independence. For this analysis, only patients transplanted between 2000 and 2018 were analyzed. We estimated the mean predicted 5 year rate of insulin independence using an online calculator based on PGF [30]. As previously outlined [30], this calculator solely depending on the value of the primary graft function was constructed and validated using a cohort of islet recipients and predicts diverse outcomes validated in ITx [17].

All statistical analyses were performed using SAS Studio Statistics (version 3.81) and Prism GraphPad (Version 10.0.1) software.

## Results

### Characteristics of the Study Population

Among 476 patients who benefited from PTx in Nantes (377 SPK/43 PAK/56 PTA), 209 recipients did not meet the inclusion criterion of the study, mainly because the lack of available HbA1c and/or C-peptide at 3 months after transplantation (*n* = 207), or because of early graft loss (*n* = 60). The individuals excluded for missing values showed no clinically relevant differences when compared to the included recipients (Supplementary Table S1). Baseline and transplantation characteristics of the study participants are described in Table 1. Of these recipients, 172 (82%) underwent SPK, 23 (11%) received PTA, and 14 (7%) received PAK transplantation.

**TABLE 1:** Recipient, graft and transplantation characteristics in the Islet transplantation cohort.

	ITA/IAK *n* = 39	PTA/PAK *n* = 37	SPK *n* = 172
Pre-transplantation recipient’s characteristics			
Female gender, n (%)	20 (51%)	18 (49%)	63 (37%)
Age (years), mean (SD)	45 (8)	42 (±9)	40 (±7)
Body mass index (kg/m^2^)	24 (±3)	25 (±4)	23 (±3)
HbA1c (%)	8.2 (±1.0)	9.4 (±2.6)	8.3 (±1.5)
Preformed donor specific antibody	1 (3%)	2 (6%)	14 (10%)
Transplantation characteristics			
Islet transplantation			
Number of islet infusions	2.7 (±0.5)		
Time between first and last infusion, months	2.7 (1.6–4.1)		
Total islet mass transplanted, 10^3^ IEQ/kg of recipient weight	13.6 (11.7–15.9)		
Total tissue volume (mL)	12.9 (9.7–14.9)		
Islet purity^a^ (%)	47 (44–54)		
Islet viability^a^ (%)	93 (91–96)		
Pancreas transplantation			
Female donor		12 (32%)	59 (34%)
Donor age (years)		32 (±14)	33 (±11)
Donor body mass index (kg/m^2^)		23 (±3)	23 (±3)
Cold ischemia time (min)		603 (±161)	658 (±177)
Immunosuppression			
T-cell depleting agent induction	11 (28%)	35 (95%)	161 (95%)
Calcineurin inhibitor	39 (100%)	37 (100%)	167 (98%)
m-TOR inhibitor	28 (72%)	37 (100%)	3 (2%)
Corticosteroid therapy	0 (0%)	32 (86%)	153 (90%)

Recipient, and transplantation characteristics are reported as n (%), mean (SD), or median (IQR) as appropriate.

^a^ The overall islet purity and viability were the weighted median (IQR) of the two or three islet infusions transplanted by the volume of each preparation. The total tissue volume was the sum of the volume of each infused preparation in the recipient.

ITA, islet transplant alone; IAK, islet after kidney; PTA, pancreas transplant alone; PAK, pancreas after kidney; SPK, simultaneous pancreas kidney; m-TOR inhibitor, mammalian target of rapamycin inhibitor.

All 39 patients who underwent ITx in Lille during the study period (16 IAK/23 ITA) were enrolled. The baseline recipient’s and transplantation characteristics are provided in Table 1. Among them, 28 (72%) received three islet cell infusions, while 11 (28%) received two infusions. A total of 106 infusions of human islets were carried out, with recipients experiencing a median overall transplantation duration of 2.7 months (IQR 1.6–4.1). There were no further infusions conducted throughout the follow-up period. The median total islet mass transplanted was 13.6 thousand islet-equivalents per kg of body weight (IQR 11.7–15.9).

A total of 56 non-transplanted normoglycemic individuals were included in this study. Of these, 43 (77%) were women, their median age was 41 (IQR 34–48) years, and their median BMI was 37.6 (IQR 27.0–38.9) kg/m^2^

### Primary Graft Function

**FIGURE 1:**
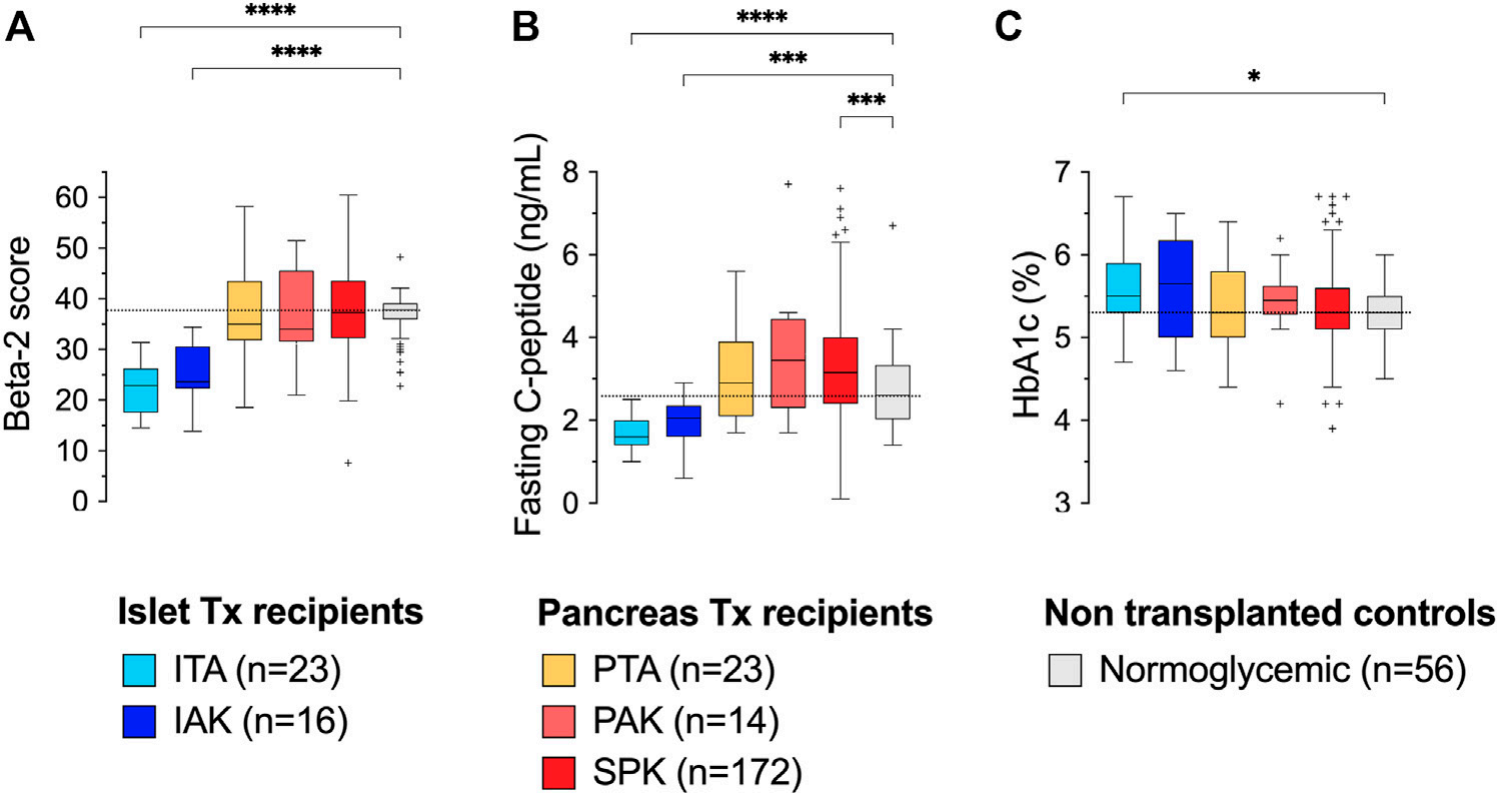
Beta-2 score **(A)**, fasting serum C-peptide **(B)** and HbA1c **(C)** values in islet recipients, pancreas recipients and non transplanted control individual The distribution is represented in the form of a box plot using the Tukey method, where the line in the middle of the box is drawn at the median, the box limits represent the 25th and 75th percentiles, and the whisker limits are represented from the value of the 25th percentile minus 1.5 times the interquartile range (IQR) to the value of the 75th percentile plus 1.5 times the IQR. Outliers are represented individually. *p* values ≤0.001 are summarized with an asterisk. Groups were compared with Welch ANOVA tests. Symbol meaning: *p* ≤ 0.05 (*); *p* ≤ 0.01 (**); *p* ≤ 0.001 (***); *p* ≤ 0.0001 (****) ITA, Islet Transplantation Alone; IAK, Islet After Kidney transplantation; PTA, Pancreas Alone; PAK, Pancreas After Kidney; SPK, Simultaneous Pancreas-Kidney.

Mean[SD] primary graft function estimated with the Beta-2 score in ITA, IAK, PTA, PAK, and SPK recipients was 22.3[5.2], 24.8[6.4], 38.9[15.3], 36.8[9.0], and 38.7[10.5], respectively. Mean beta-cell function estimated with the Beta-2 score in normoglycemic controls was 36.6[4.3]. As displayed in Figure 1A, the mean values of primary graft function in ITA and IAK recipients were significantly lower than the mean beta-cell function measured in normoglycemic controls (*p* < 0.0001). Conversely, the mean value of primary graft function in PTx recipients with a surviving graft and the mean beta-cell function measured at the time of enrolment in normoglycemic controls were similar.

The mean fasting C-peptide levels were significantly higher in the controls compared to ITA (*p* < 0.0001) and IAK (*p* = 0.001), but they were significantly lower compared to SPK (*p* < 0.0001) and similar to those of PTA (*p* = 0.643) and PAK (*p* = 0.310) (Figure 1B).

Of note, the overall HbA1c values of normoglycemic controls did not significantly differ from those in IAK, PTA, PAK and SPK recipients but were significantly lower compared to ITA (*p* = 0.191) (Figure 1C).

### Five-Year Insulin Independence

Among the 39 islet-transplanted recipients, two never achieved insulin independence, and 22 patients (56.4%) were not insulin independent at 5 years. At the last follow-up, 32 ITx recipients out of 39 had a functional graft (serum C-peptide ≥0.3 ng/mL). Among the 209 recipients who received PTx and had a functional pancreas graft at 3 months, 23 patients (11.0%) had lost insulin independence during the 5 years follow-up. Of note, 12.5% of the overall cohort of PTx recipients experienced a graft loss before 3 months and were therefore excluded from the present analysis (60 out of 476 pancreas recipients).

### Relation Between Primary Graft Function and 5 Year Outcome

**FIGURE 2:**
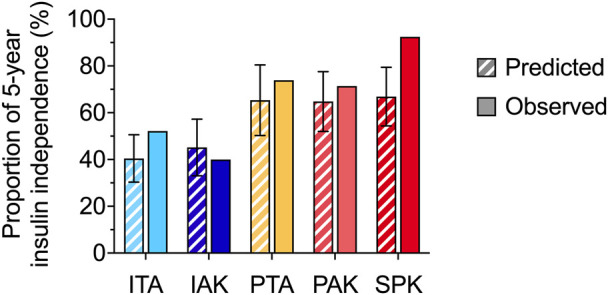
Predicted and observed proportion of 5 years insulin independence among islet and pancreas recipient with initial graft function The mean (95% confidence interval) of predicted 5 year rate of insulin independence (hatched bar) and the observed 5 year rate of insulin independence (solid bar) are reported for the various recipient subgroups. IA, Islet Transplantation Alone; IAK, Islet After Kidney transplantation; PA, Pancreas Alone; PAK, Pancreas After Kidney; SPK, Simultaneous Pancreas-Kidney.

We used the PGF-based calculator available online [30] to estimate the mean (95% CI) proportion of patients in each subgroup with 5 year insulin independence, as illustrated in Figure 2, the proportion of insulin-independent patients observed at 5 years remained within the prediction confidence interval determined by the calculator in islet and solitary pancreas recipients but not in SPK. Indeed, in this subgroup of patients, the observed rate of 5 year insulin-independence was significantly higher than the rate predicted with the PGF-based calculator.

## Discussion

In the current study, we analyzed the early post-transplant beta-cell function, referred to as primary graft function, in islet transplantation and pancreas transplantation, and examined its relationship with the 5 year rate of insulin independence across all transplantation modalities.

Our study demonstrated that primary graft function values were comparable between ITA and IAK, as well as between PTA, PAK, and SPK. However, mean value of primary graft function was significantly lower in ITx recipients compared to PTx recipients. Primary graft function values in ITx recipients were also significantly lower than the beta-cell function observed in normoglycemic controls. In contrast, pancreas transplant recipients exhibited primary graft function values similar to beta-cell function values in normoglycemic controls.

Notably, serum C-peptide levels in normoglycemic controls were higher than in the ITA and IAK groups. However, these levels were similar to those in solitary pancreas recipients but lower than in SPK recipients. Every islet and pancreas recipient exhibited marked improvements in HbA1c levels, aligning with the American Diabetes Association’s recommended targets, compared to their pre-transplant values. Additionally, mean HbA1c values were not significantly different between the various types of transplantation, except for ITA, where recipients exhibited significantly higher values.

Secondly, our findings indicated that for PTA and PAK recipients, the calculator’s predictions of 5 year insulin independence rates, which were based solely on primary graft function, were relatively precise. In contrast, the calculator tended to underestimate the outcomes in SPK recipients.

These results are in line with a recent study demonstrating the independent linear association between primary graft function and various 5 year outcomes of ITx [17], including graft function, insulin independence, adequate glucose control, and overall transplantation success assessed with the Igls 2.0 criteria [31]. To our knowledge, the present study is the first to extend these results in the context of PTx. These findings indicate that the difference in long-term outcomes of PTx and ITx are likely attributable to the superior initial function of islets that survive the transplantation of a vascularized pancreas, in contrast to isolated islets infused in the portal vein. Of note, all subgroups transplanted with a vascularized pancreas had similar primary graft function. The 5 year insulin independence rate observed in patients who simultaneously received a kidney from the same donor (SPK) was, however, superior than in those who received a solitary pancreas (PTA/PAK). This difference between SPK and solitary pancreas transplant was also reported in the International Pancreas Transplant Registry and related to the reduction of immunologic graft loss [32]. In a study on SPK recipients, synchronous pancreas and kidney rejection occurred in 73%, kidney-only rejection occurred in 23% and pancreas-only rejection occurred in only 3% of biopsies [33]. Taken together, these results suggest a positive impact of monitoring kidney function for early detection and treatment of the overall allogenic immune response. Diagnosing immune rejection remains therefore challenging in solitary pancreas or islet transplantation [34].

Several limitations need to be considered when interpreting our study. First, the retrospective design of the study and the limited sample size for certain groups could have introduced selection bias. A prospective study in a larger cohort of patients could yield more robust and generalizable results. Additionally, data were collected from two parallel single center cohorts, which could introduce variations in patient selection and follow-up protocols. Multicenter studies with standardized protocols could help mitigate this potential bias and strengthen the study’s findings.

Second are the method and timing used to estimate primary graft function. Several composite indexes have been proposed to estimate beta-cell function [35]. We chose here to use the Beta-2 score, a simple and continuous score validated in ITx [24]. The use of more sophisticated tests to estimate primary graft function, such as dynamic tests of insulin secretory reserve, could have refined the prediction of long-term outcomes [36]. As previously described, primary graft function was assessed 1 month after the last islet infusion [17], which corresponds to the necessary time for full revascularization of islets transplanted in the liver [37]. In practice, this also resulted in a mean duration of 4.3 [2.8] months after the first islet infusion. The optimal timing for assessing PGF after the transplantation of a vascularized pancreas is unknown. Here, we used 3 months for this was the earliest data available in the study’s participants.

Of note, PTx recipients who experienced graft failure before that date (60 cases) had to be excluded from this retrospective study, since 5 year follow-up data were not available, resulting in a twelve percent overestimation of the reported 5 year rate of insulin independence after PTx. This exclusion of early pancreatic graft failures may be debatable. However, since our main objective was to evaluate the predictive value of early beta-2 score in functional pancreas transplant recipients for long-term graft function, we assume this exclusion did not introduce bias into our study’s analysis and conclusions. It is also important to note that half of the eligible pancreas transplant recipients were excluded from the analysis due to missing data. Nonetheless, as the included and excluded groups were comparable (Supplementary Materiel), we assume these exclusions did not introduce bias into our analysis.

Finally, it should be noted that organ allocation rules differ for islet and pancreas recipients in France and many countries. This practically favors the use of organs from donors with lower BMI and younger age in pancreas Tx. This potential selection bias, may have contributed to higher primary graft function observed in pancreas Tx recipients.

In summary, this study showed that the beta cell function restored in patients with Type 1 diabetes following islet Tx, even after multiple infusions, remains generally inferior to the levels observed in recipients of pancreas Tx and to those measured in control individuals. Our results also suggest that this difference in PGF likely explains the difference in 5 year rate of insulin independence generally observed between islet and pancreas Tx. Overall, this study suggests for the first time, a potential use of primary graft function as an early predictor of long-term outcome of PTx, principally PTA and PAK. Optimal primary graft function indicates better graft function and a higher likelihood of maintaining long-term insulin independence. However, to better understand this predictive role, further research is needed in the context of PTx. Prospective, larger scale, long-term studies remain warranted to distinguish the respective role of primary graft function and confounding factors, such as recipient allogeneic and autoimmune reactions, and the effects of immunosuppressive treatments.

In conclusion, the present study supports the value of primary graft function in the management of type 1 diabetes patients undergoing beta-cell replacement with various modalities, such as PTx, ITx, or other insulin-secreting cell transplantation.

## Data Availability

Datasets presented in this article are available upon reasonable request to corresponding authors.

## Abbreviations

PGF, Primary Graft Function; T1D, Type 1 Diabetes; PTx, Pancreas transplantation; ITx, Islet transplantation; ITA, Islet Transplantation Alone; IAK, Islet After Kidney transplantation; PTA, Pancreas Transplantation Alone; PAK, Pancreas After Kidney; SPK, Simultaneous Pancreas-Kidney.
