# Supplementary material for: Primary Graft Function and 5 Year Insulin Independence After Pancreas and Islet Transplantation for Type 1 Diabetes: A Retrospective Parallel Cohort Study
Source: Transpl Int. 2023 Dec 28;36:11950. doi: 10.3389/ti.2023.11950 (PMC10783428; doi:10.3389/ti.2023.11950)
Supplement: Supplementary file 1 [file Table1.DOCX]

**Supplementary Table S1 : Functional pancreas allograft at 3 months post-transplant: included versus excluded recipients based on Primary Graft Function availability**

|  | **All (n=416)** | | | **Included in**  **analysis (n=209)** | | | **Excluded from analysis (n=207)** | | | **p-value** |
| --- | --- | --- | --- | --- | --- | --- | --- | --- | --- | --- |
|  | **NA** | **n** | **%** | **NA** | **n** | **%** | **NA** | **n** | **%** |  |
| Pancreas recipients |  |  |  |  |  |  |  |  |  |  |
| SPK | 0 | 330 | 79.3 | 0 | 172 | 82.3 | 0 | 158 | 76.3 | 0.147 |
| PAK | 0 | 37 | 8.9 | 0 | 14 | 6.7 | 0 | 23 | 11.1 | 0.121 |
| PTA | 0 | 49 | 11.8 | 0 | 23 | 11.0 | 0 | 26 | 12.6 | 0.651 |
| Re-transplantation | 0 | 47 | 11.3 | 0 | 18 | 8.6 | 0 | 19 | 9.1 | > 0.999 |
| Male donor | 0 | 260 | 62.5 | 0 | 138 | 66.0 | 0 | 122 | 58.4 | 0.130 |
| Vascular cause of donor’s death | 1 | 178 | 42.9 | 0 | 84 | 40.2 | 1 | 94 | 45.6 | 0.234 |
| Donor’s history of hypertension | 23 | 24 | 6.1 | 9 | 12 | 6.0 | 14 | 12 | 6.2 | > 0.999 |
| Donor’s history of cardiac arrest | 4 | 82 | 19.9 | 1 | 47 | 22.6 | 3 | 35 | 17.1 | 0.177 |
| Use of vasoactive drugs in the donor | 23 | 337 | 85.7 | 11 | 176 | 88.9 | 12 | 161 | 82.6 | 0.083 |
| Male recipient | 0 | 246 | 59.1 | 0 | 128 | 61.2 | 0 | 118 | 56.5 | 0.425 |
| Immunosuppressive regimen at 3 months |  |  |  |  |  |  |  |  |  |  |
| Depletant induction | 2 | 397 | 95.9 | 2 | 196 | 94.7 | 0 | 201 | 97.1 | 0.322 |
| Calcineurin inhibitors | 17 | 380 | 95.3 | 0 | 200 | 95.7 | 17 | 180 | 94.7 | 0.189 |
| mTor inhibitors | 17 | 49 | 12.2 | 0 | 25 | 11.9 | 17 | 24 | 12.6 | 0.879 |
| Antiproliferative drugs | 17 | 362 | 90.7 | 0 | 190 | 90.9 | 15 | 172 | 89.6 | 0.737 |
| Steroids | 15 | 165 | 41.3 | 0 | 86 | 41.1 | 15 | 79 | 39.9 | > 0.999 |
|  |  |  |  |  |  |  |  |  |  |  |
|  | **NA** | **mean** | **SD** | **NA** | **mean** | **SD** | **NA** | **mean** | **SD** |  |
| Donor age (years) | 0 | 33.5 | 11.0 | 0 | 32.6 | 11.4 | 0 | 34.5 | 10.6 | 0.084 |
| Donor BMI (kg/m^2^) | 0 | 22.8 | 2.8 | 0 | 23 | 2.7 | 0 | 22.6 | 2.9 | 0.084 |
| Pancreas cold ischemia time (min) | 13 | 697 | 179 | 3 | 648 | 175 | 10 | 748 | 169 | < .0001 |
| Recipient age (years) | 0 | 40.3 | 7.8 | 0 | 40.2 | 7.4 | 0 | 40.3 | 8.2 | 0.863 |
| Recipient BMI (kg/m^2^) | 0 | 23.1 | 3.4 | 0 | 23.6 | 3.5 | 0 | 22.6 | 3.3 | 0.002 |
| Primary graft function (Beta2-score at 3 months) | 207 | 41.7 | 30.9 | 0 | 38.6 | 10.9 | 206 | - | - | - |
| Fasting blood glucose (mmol/l) | 40 | 5.2 | 0.9 | 0 | 5.2 | 0.9 | 40 | 5.2 | 0.9 | 0.217 |
| Fasting C-peptide (ng/ml) | 72 | 3.4 | 1.8 | 0 | 3.5 | 1.8 | 72 | 3.4 | 1.8 | 0.357 |
| HbA1c (%) | 199 | 5.3 | 0.5 | 0 | 5.3 | 0.5 | 199 | 5.3 | 1.1 | 0.137 |
| Exogenous insulin needs (UI/mL/kg) | 0 | 0 | 0 | 0 | 0 | 0 | 0 | 0 | 0 | 1.000 |

SPK : Simultaneous Pancreas Kidney; PAK : Pancreas After Kidney; PTA : Pancreas Transplant Alone; BMI : Body Mass Index; NA: non available data
